# Enhancing Transdermal Delivery: The Role of Gecko-Derived Cathelicidin Peptide G3CY-10 in UV-Induced Skin Photoaging

**DOI:** 10.3390/biom15111515

**Published:** 2025-10-27

**Authors:** Yunjiao Wang, Zicheng Ma, Fengshuo Li, Xuanzeng Li, Ningyang Gao, Junhan Wang, Shasha Cai

**Affiliations:** The Key Laboratory for Medical Functional Nanomaterials, College of Medical Engineering, Jining Medical University, Jining 272067, China; wangyunjiaow@gmail.com (Y.W.); mamazicheng1@gmail.com (Z.M.); fengshuolf@gmail.com (F.L.); piaoluop@gmail.com (X.L.); gaoningyang23@gmail.com (N.G.); junhanwang548@gmail.com (J.W.)

**Keywords:** skin photoaging, cathelicidin, microemulsion gel, oxidative stress, collagen degradation

## Abstract

Ultraviolet (UV) radiation-induced skin photoaging impacts both appearance and skin health, potentially leading to disorders and cancer. Unlike traditional sunscreens, natural antioxidants can target photoaging at its source. Among these, cathelicidins have attracted considerable research interest due to their multifunctional properties. This study examines the gecko-derived cathelicidin-modified peptide G3CY-10, utilizing a microemulsion gel delivery system to address the challenges related to the transdermal absorption of macromolecular peptides, and systematically assesses its anti-photoaging effects and underlying mechanisms. The results demonstrate that the G3CY-10 microemulsion, formulated with a lecithin–ethanol–butyl acetate system (km = 1:1), exhibits notable stability, and the gelation significantly enhances the transdermal delivery efficiency of G3CY-10. The anti-photoaging efficacy of the G3CY-10 microemulsion gel is substantiated by its capacity to mitigate UV-induced skin photoaging in murine models. This is evidenced by a decrease in epidermal thickness, suppression of sebaceous gland proliferation, and restoration of collagen fiber density. Masson staining further corroborates a significant reduction in collagen degradation. Mechanistic analyses suggest that G3CY-10 primarily confers protection by inhibiting UV-induced collagen degradation and reversing the depletion of superoxide dismutase. This study provides a theoretical foundation and technical support for the clinical translation of natural peptides and the development of innovative anti-photoaging products.

## 1. Introduction

Over the past two decades, the annual depletion of the global ozone layer and the continuous expansion of the ozone hole have exacerbated the detrimental effects of UV radiation on human health. Studies have indicated that over 80% of facial aging is attributable to chronic UV radiation exposure [1,2]. Skin damage caused by environmental factors, particularly solar UV radiation, is referred to as photoaging [2]. Beyond contributing to skin aging, photoaging can result in various dermatological conditions, including actinic keratosis, nodular elastosis, and melanocytic tumors. Consequently, the prevention and treatment of skin photoaging have become critical challenges.

Antioxidants present a more effective approach than sunscreens for tackling the underlying causes of skin photoaging [3]. Recently, natural antioxidants, especially peptides, have gained attention in treating skin photoaging. Glutathione and carnosine are frequently used in sunscreens and anti-aging products. Certain small peptides with antioxidant properties have been integrated into repair solutions and eye creams to promote collagen synthesis and reduce the appearance of wrinkles. However, the transdermal delivery of antioxidant peptides with larger molecular structures remains challenging due to low bioavailability [4]. The skin’s low permeability limits the penetration of most pharmaceutical molecules, and the large molecular size, hydrophilicity, and charge of antioxidant peptides further impede their ability to penetrate the stratum corneum. Additionally, the thickening of the epidermis and the increased formation of wrinkles, both resulting from photo-aging, further obstruct the transdermal absorption of these compounds. Therefore, improving skin permeability and enhancing the bioavailability of peptides are critical challenges that need to be addressed for the effective application of antioxidant peptides in the prevention and treatment of skin photo-aging.

In recent years, microemulsions have gained prominence in peptide drug formulations due to their numerous advantages, such as ease of preparation, high solubilization capacity, efficient drug dispersion, and stability without compromising the biological activity of peptides [5,6]. Szumala et al. demonstrated that microemulsions can enhance the dermal penetration of macromolecules, including hyaluronic acid and collagen, thereby promoting the improvement and regeneration of aging and diseased skin [6]. Similarly, Rastogi et al. successfully employed microemulsions for the transdermal delivery of T4 phages, targeting drug-resistant bacterial infections [7]. Despite these advancements, challenges remain in formulating microemulsions as carriers for the transdermal delivery of antioxidant peptides. These challenges include insufficient adhesion, difficulties in application and retention on the skin, and the tendency for water evaporation during extended storage. The gelation of microemulsions effectively addresses these issues and has become a central focus in research on transdermal drug delivery systems. Organic gels derived from microemulsions have been shown to significantly enhance adhesion and storage stability, while also improving local efficacy and facilitating transdermal drug transport [8].

Cathelicidins represent a crucial family of host defense peptides present in vertebrates, playing a significant role in modulating the innate immune response. In recent years, the non-antibacterial functions of cathelicidins have garnered increasing scholarly attention worldwide, particularly concerning their involvement in skin injury repair. For instance, Cathelicidin-OA1, derived from the Yunnan odorous frog, has been shown to facilitate skin wound healing by inducing the release of TGF-β1 [9]. Similarly, Cathelicidin-NV from the abdominal-spotted tree frog has been demonstrated to enhance the proliferation of human skin fibroblasts and immortalized keratinocytes, accelerate the healing of full-thickness skin wounds in murine models, and offer protective effects against UVB-induced skin photoaging [10]. Despite these advancements, a significant research gap persists regarding the application of cathelicidins from the Reptilia class in the treatment of skin injuries.

Our research group has previously shown that the antioxidant peptide Gj-CATH3, along with its modified derivative G3CY-10, derived from *Gekko japonicus*, exhibits substantial antioxidant properties and facilitates the repair of dermal wounds in murine models [11]. In the current study, we have developed a microemulsion gel delivery system specifically for the modified peptide G3CY-10, evaluated its efficacy in mitigating photoaging, and elucidated the underlying mechanisms. This investigation aims to enhance the current understanding of cathelicidin functions, provide theoretical and technical foundations for the clinical application of gecko-derived compounds, and contribute to the development of anti-photoaging products, thereby holding substantial public health implications.

## 2. Materials and Methods

### 2.1. Peptide Synthesis

G3CY-10 (CGWKHCGWKY) was synthesized by GL Biochem Co., Ltd. (Shanghai, China). The synthetic peptides were analyzed using high-performance liquid chromatography (HPLC) and matrix-assisted laser desorption ionization time-of-flight (MALDI-TOF) mass spectrometry to confirm a purity greater than 96%.

### 2.2. Preparation of Blank Microemulsions

Microemulsions were synthesized utilizing the water-in-oil technique, incorporating lecithin, Span 80, and Tween 80 as surfactants, anhydrous ethanol as a cosurfactant, and a combination of castor oil and ethyl butyrate as the oil phase. An initial screening of formulations was performed to evaluate the feasibility of successful microemulsion formation. The mass ratio of surfactant to cosurfactant (Km) was consistently maintained at 3:1. During this preliminary assessment, surfactants were evaluated for their efficacy in forming microemulsions. The mixed surfactants and oil phase were blended in mass ratios of 0:10, 1:9, 2:8, 3:7, 4:6, 5:5, 6:4, 7:3, 8:2, 9:1, and 10:0. Subsequently, blank microemulsions were prepared using the water titration method [12]. A pseudo-ternary phase diagram was constructed from these preparations, with the selection of the appropriate surfactant being guided by the evaluation of the area size within the phase diagram [13]. Following the identification of the optimal surfactant combination—utilizing anhydrous ethanol as a cosurfactant and ethyl butyrate as the oil phase—various Km values (4:1, 3:1, 2:1, and 1:1) were tested to determine which configuration yielded the largest pseudo-ternary phase diagram.

### 2.3. Identification and Evaluation of Microemulsion Type and Quality

Building on the optimal microemulsion formulation outlined in Section 2.2, microemulsions containing G3CY-10 at a concentration of 40 μM were synthesized for further analysis. The type of microemulsion was identified using staining techniques, which involved evaluating the diffusion rates of Sudan Red and Methylene Blue within each formulation [14].

To assess physical stability, the microemulsion was subjected to centrifugation, facilitating the observation of phenomena such as layering, flocculation, demulsification, and turbidity [15]. Particle size analysis was performed using transmission electron microscopy (TEM). For this analysis, one or two drops of the diluted microemulsion sample were carefully placed onto a copper grid using a dropper and allowed to stand for 10 min. The supernatant was then removed with filter paper, and a 2% phosphotungstic acid solution was applied for staining. After the drying process, the copper grid was examined using a Tecnai 12 transmission electron microscope operating at 120 kV (Philips, Amsterdam, The Netherlands) to assess both the particle size and morphology of the microemulsion. The particle size data from the microemulsion samples were statistically analyzed using Image J software v1.8.0.

### 2.4. Preparation of Microemulsion Gel

The microemulsion gel was synthesized utilizing the swelling diffusion method. Based on our preliminary optimization studies, a specified amount of G3CY-10 microemulsion was uniformly coated with Pluronic F407 (P407) powder to achieve a final concentration of 4%. This mixture was thoroughly blended and allowed to swell completely at ambient temperature for a duration of 24 h. Subsequently, the mixture was homogenously stirred in a water bath maintained at 25 °C to generate the G3CY-10 microemulsion gel. The physical appearance of the gel was meticulously observed and documented.

### 2.5. Ethical Considerations

All animal experiments were conducted in accordance with the Guidelines for the Care and Use of Laboratory Animals (NIH Publications No. 8023, revised 1987) and received approval from the Animal Studies Local Ethics Committee of Jining Medical University (approval number 2022-DW-148).

### 2.6. Effects of Microemulsion Gel on the Transdermal Delivery of G3CY-10

To assess the transdermal effects, a microemulsion gel containing 1 mM fluorescein isothiocyanate (FITC)-labeled G3CY-10 was formulated [16]. A total of forty-two female ICR mice, with body weights ranging from 14 to 18 g, were randomly divided into two distinct groups (n = 21). The dorsal hair of these mice was removed using depilatory cream one day prior to the initiation of the experiment. In a light-proof environment, 100 μL of FITC-labeled G3CY-10 microemulsion gel was applied to the hairless dorsal regions of each mouse, while an equivalent volume of FITC-labeled G3CY-10 aqueous solution was used as a control treatment. Three mice were euthanized at various time points, specifically at 0.5, 1, 2, 3, 4, 8, and up to 16 h post-administration. The dorsal skin was excised, embedded, and subjected to frozen sectioning. The sections were scanned using a Pannoramic Midi digital slide scanner (3DHISTECH Ltd., Budapest, Hungary) and analyzed with CellQuant software v2.4.0.119028 (3DHISTECH Ltd., Budapest, Hungary).

### 2.7. Mouse Model of Skin Photoaged and Peptide Treatment

**Figure 1:**
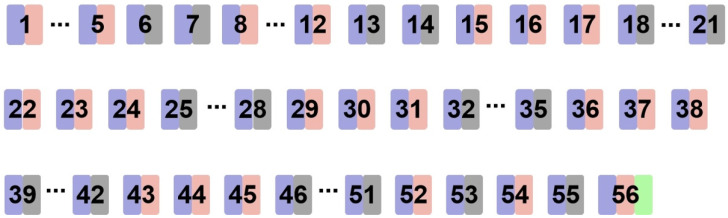
Timeline of the treatment procedure in animal experiments. Purple indicates the administration of a 40 μM drug treatment on the experimental day. Pink represents UV irradiation treatment. Gray represents no UV irradiation treatment on the experimental day. Green signifies the conclusion of the experiment.

Adult female ICR mice (n = 35, average weight 14 g) were procured from Xingkang Biotechnology Co., Ltd., located in Jinan, China. The mice were allowed a 7-day acclimatization period before commencing the experimental procedures. Subsequently, the mice were randomly assigned to four groups, each consisting of seven individuals: the Sham group (untreated mice without UV exposure), the PBS group (mice treated with PBS in microemulsion gel following UV exposure), the G3CY-10 group (mice treated with 40 μM G3CY-10 in microemulsion gel post-UV exposure), and the antioxidant vitamin C (Vc) group (mice treated with 40 μM Vc in microemulsion gel post-UV exposure) [17]. UV irradiation was administered using a full-spectrum sunlight simulation lamp (ULTRA VITALUX, OSRAM, Munich, Germany) with a wavelength range of 280–400 nm. Prior to treatment, the mice were anesthetized with isoflurane, and their dorsal hair was removed using a razor and VEET hair removal cream. The mice were then placed in a specialized cage and subjected to UV radiation at an intensity of 1 minimal erythema dose (MED) five times during the first week, followed by 2 MED three times per week for a duration of seven weeks (refer to Figure 1). The intensity of UV irradiation was measured using a UV LIGHT METER (Lutron UV-340A, Taiwan, China). Following the completion of the study, the mice were sacrificed for further analysis. The skin tissues were snap frozen in liquid nitrogen, and others were fixed in 4% paraformaldehyde for use.

### 2.8. Histomorphological Analysis

Hematoxylin and eosin (H&E) staining and Masson staining were carried out for histomorphological analysis. The fixed skin tissues were embedded in paraffin and sectioned into slices of 4 μm thickness using a LEiCA RM2016 microtome (Leica, Shanghai, China). Following mounting on slides, the sections underwent deparaffinization and hydration processes. H&E staining and Masson’s trichrome staining (Powerful Biology Co., Ltd., Wuhan, China) were performed according to the manufacturer’s protocol. The quantitative analysis of Masson staining was determined using Image J software.

### 2.9. Immunohistochemistry (IHC)

Tissues were fixed, embedded in paraffin, and sectioned into continuous slices of 5 μm thickness. The paraffin sections were subjected to deparaffinization and rehydration. Antigen retrieval was performed by heating the sections in sodium citrate buffer using a microwave. Subsequently, the sections were washed with phosphate-buffered saline (PBS) for 5 min, repeated three times at room temperature, and incubated in 3% hydrogen peroxide (H_2_O_2_) for 25 min at room temperature. Following another series of PBS washes for 5 min, repeated three times, the sections were blocked and incubated in 3% bovine serum albumin (BSA) for 30 min. The primary antibody, anti-collagen I mouse antibody (Abcam, Cambridge, MA, USA), was applied dropwise, and the sections were incubated at 4 °C overnight. On the subsequent day, the primary antibody was removed, and the sections were washed with PBS for 5 min, repeated three times. After a 50 min incubation with a goat anti-mouse secondary antibody (Abcam, Cambridge, MA, USA), the sections were again washed with PBS for 5 min, repeated three times. Finally, freshly prepared diaminobenzidine (DAB) was added for color development, and the sections were counterstained with hematoxylin (Solarbio, Beijing, China), dehydrated, and mounted with neutral gum. All slides were scanned using a Pannoramic Midi digital slide scanner (3DHISTECH Ltd., Budapest, Hungary) and analyzed using CellQuant software v2.4.0.119028 (3DHISTECH Ltd., Budapest, Hungary).

### 2.10. Detection of Skin Inflammation-Related Indicators

The dorsal hair of mice was removed, and the skin tissue was rinsed with pre-cooled PBS to remove subcutaneous fat and connective tissue. After blotting dry with filter paper, the tissue was placed into a 1.5 mL centrifuge tube and weighed. The tissue was then minced using ophthalmic scissors, and 3–5 small steel beads were added. PBS was added at a weight-to-volume ratio of 1:9, and the mixture was homogenized in a frozen tissue grinder (SCIENTZ-48L, Scientz Biotechnology Co., Ltd., Ningbo, China) for 10 min. Following centrifugation, the supernatant was collected and stored for subsequent analysis. The level of IL-6 in the skin tissue homogenate was quantified using enzyme-linked immunosorbent assay (ELISA) kit (ELabsciecne, Wuhan, China), strictly following the manufacturer’s instructions.

### 2.11. Detection of Antioxidant Indicators in Skin Tissue

Mouse skin homogenates were prepared according to the kit instructions, followed by centrifugation. The supernatants were used to measure the activity of superoxide dismutase (SOD, Beyotime Biotechnology, Shanghai, China) and the content of malondialdehyde (MDA, Beyotime Biotechnology, Shanghai, China), respectively.

### 2.12. Determination of Collagen Content in Skin

Hydroxyproline (Hyp) is a characteristic amino acid found specifically in mammalian collagen. The total collagen content can be estimated by multiplying the Hyp content by a factor of 7.46 [18]. Mouse skin samples were hydrolyzed in alkaline hydrolysis solution at 95 °C for 20 min. The pH was then adjusted to neutrality, and the sample was centrifuged to collect the supernatant. The collagen content was quantified according to the manufacturer’s instructions (Nanjing Jiancheng Bioengineering, Nanjing, China).

### 2.13. Data Analysis

All statistical analyses were performed using GraphPad Prism V.5.0 software (GrapdPad Software Inc., SanDiego, CA, USA). Unpaired Student’s *t*-tests were conducted to compare the two groups. Two-tailed *p* < 0.05 (*) and *p* < 0.01 (**) signified statistical significance. All the results were reported as mean ± SEM in three independent experiments.

## 3. Results

### 3.1. Preparation and Quality Assessment of the G3CY-10 Microemulsion Gel Delivery System

The results of the initial screening experiment revealed that the combinations of oil phase and surfactant, specifically lecithin/castor oil and Span80/ethyl butyrate, with anhydrous ethanol serving as the co-surfactant, were ineffective in forming stable microemulsions (Table 1). Consequently, four alternative combinations were selected for further formulation optimization: ethyl butyrate/lecithin/anhydrous ethanol, ethyl butyrate/ Tween80/anhydrous ethanol, castor oil/Span80/anhydrous ethanol, and castor oil/Tween80/anhydrous ethanol.

**Figure 2:**
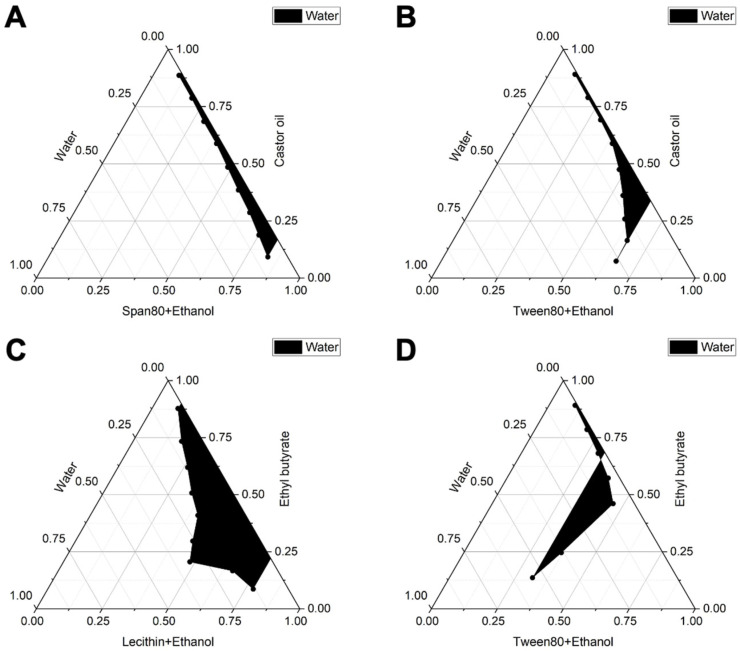
Pseudoternary phase diagrams of the oil-surfactant-water systems with a Km value of 3:1. The shaded region indicates the presence of nanoemulsions. (**A**) Castor oil as the oil phase, Span 80 as the surfactant, and ethanol as the co-surfactant; (**B**) Castor oil as the oil phase, Tween 80 as the surfactant, and ethanol as the co-surfactant; (**C**) Ethyl butyrate as the oil phase, Lecithin as the surfactant, and ethanol as the co-surfactant; (**D**) Ethyl butyrate as the oil phase, Tween 80 as the surfactant, and ethanol as the co-surfactant.

**Figure 3:**
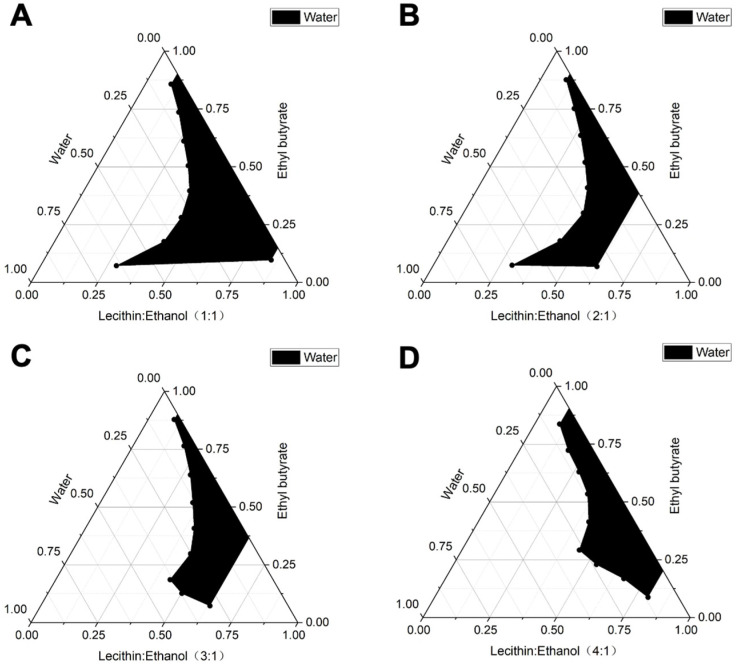
The pseudo-ternary phase diagrams are depicted at surfactant/co-surfactant mixing ratios of (**A**) 1:1, (**B**) 2:1, (**C**) 3:1, and (**D**) 4:1. Ethyl butyrate as the oil phase, Lecithin as the surfactant, and ethanol as the co-surfactant. The shaded regions in these diagrams denote the formation of nanoemulsions.

The surfactant screening results indicated that, with a fixed Km value of 3:1, the combination of lecithin as the surfactant, anhydrous ethanol as the co-surfactant, and ethyl butyrate as the oil phase yields the largest pseudo-ternary phase diagram area (Figure 2). Consequently, lecithin and ethyl butyrate were selected as the surfactant and oil phase, respectively, for the preparation of microemulsions (Figure 2C). Subsequently, the effect of varying Km values on the water-loading capacity of the microemulsion was investigated. As depicted in Figure 3, when the Km value was 1:1 and the mass ratio of mixed surfactant to oil phase was 4:1, the pseudo-ternary phase diagram area reached its maximum. Based on this formulation, the G3CY-10 microemulsion was prepared, exhibiting a water-carrying capacity of 60%.

**Figure 4:**
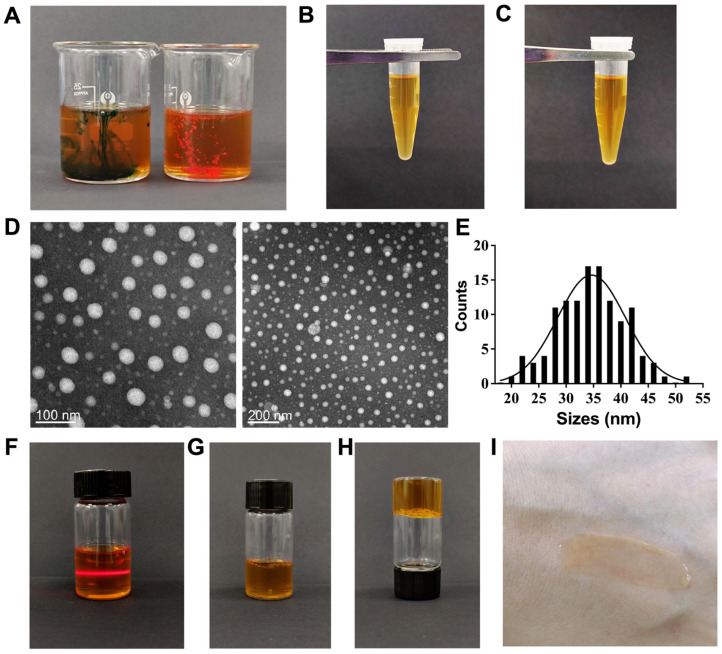
The type identification and quality evaluation of G3CY-10 microemulsion. (**A**) Microemulsion type identification using Sudan red and methylene blue staining. (**B**,**C**) Centrifugal stability analysis, with (**B**) representing the state before centrifugation and (**C**) after centrifugation. (**D**,**E**) Transmission electron microscopy analysis of the G3CY-10 microemulsion, with (**E**) providing a statistical analysis of particle size based on the results from (**D**). (**F**) The Tyndall effect in G3CY-10 microemulsion. (**G**,**H**) The depiction of G3CY-10 microemulsion in a gelled state. (**I**) Analysis of the skin spreadability of G3CY-10 microemulsion gel.

The type of microemulsion was identified using Sudan Red and Methylene Blue staining techniques. As illustrated in Figure 4A, Methylene Blue rapidly permeated the microemulsion and achieved uniform mixing within the system, whereas Sudan Red displayed limited diffusion. This observation suggests that the prepared microemulsion is of the oil-in-water (O/W) type. Quality assessment outcomes indicated that, following centrifugation at 4000 rpm for 15 min, there was no evidence of phase separation, flocculation, demulsification, or turbidity (Figure 4C), thereby affirming the microemulsion’s robust physical stability. Particle size is a critical factor affecting in vitro drug release and transdermal permeation rates in microemulsion gel systems. Previous research has demonstrated that smaller microemulsion particle sizes enhance drug penetration through the skin barrier and increase the release rate [19]. TEM analysis revealed that the G3CY-10 microemulsion exhibited a uniform spherical morphology (Figure 4D), with an average particle size of 34.59 nm (Figure 4E), which is consistent with the typical characteristics of microemulsions. The Tyndall effect, a characteristic phenomenon of colloidal systems resulting from light scattering by particles within the size range of 1–100 nm, was distinctly observed when the microemulsion was exposed to laser illumination (Figure 4F). This observation further corroborates that the G3CY-10 microemulsion exhibits typical colloidal particle size characteristics.

The G3CY-10 microemulsion gel was formulated using 4% P407 as the gel base. As depicted in Figure 4G–I, the resulting microemulsion gel was yellowish-brown, clear, and transparent, possessing a uniform and fine texture, good spreadability, low fluidity, no irritation, and strong adhesion to the skin, rendering it suitable for topical application.

### 3.2. Evaluation of the Transdermal Enhancement Effects of G3CY-10 Microemulsion Gel

**Figure 5:**
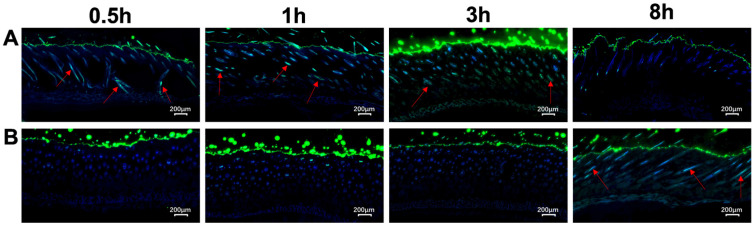
The study of the capacity of microemulsion gel to enhance the transdermal penetration of G3CY-10. (**A**) FITC labeled-G3CY-10 within microemulsion gel; (**B**) FITC labeled-G3CY-10 in an aqueous solution. The green fluorescence indicates the presence of FITC-labeled G3CY-10 peptide, and the blue DAPI staining highlights the nuclei. The red arrow signifies the transdermal entry of FITC-labeled G3CY-10 into skin tissue. The scale bar represents 200 μm.

In vivo transdermal analysis demonstrated that the FITC-G3CY-10 aqueous solution did not penetrate into subcutaneous tissue after 3 h and only reached the epidermis and dermis after 8 h, exhibiting limited penetration (Figure 5A). In contrast, the FITC-G3CY-10 microemulsion gel penetrated the epidermis and dermis within 0.5 h, reached the subcutaneous tissue within 3 h, and was fully absorbed by 8 h (Figure 5B). These findings suggest that this microemulsion gel formulation significantly enhances the transdermal delivery of G3CY-10.

### 3.3. Study on the Anti-Skin Photoaging Effects of G3CY-10

**Figure 6:**
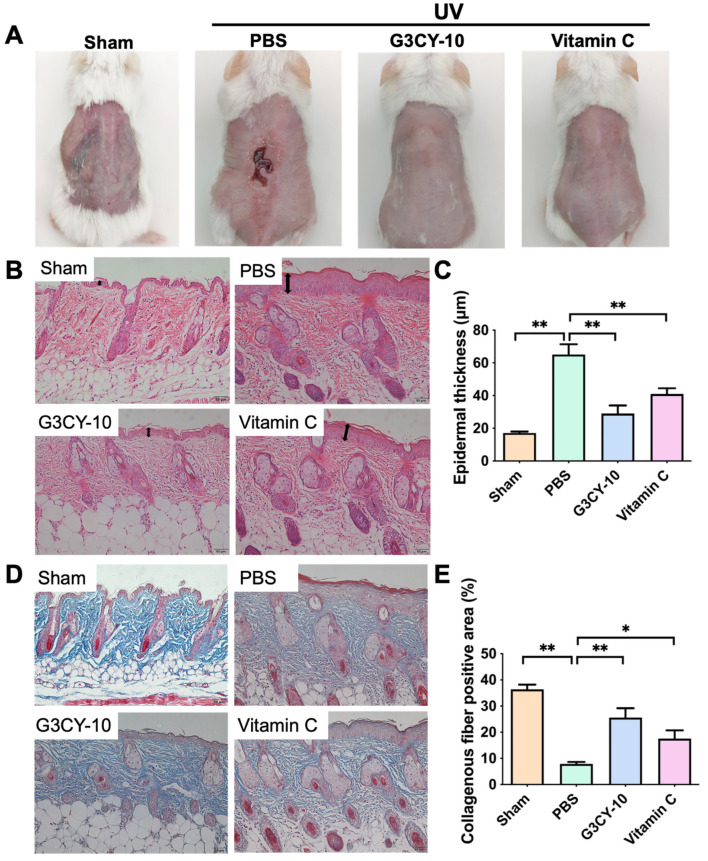
The anti-photoaging effects of G3CY-10 on the skin. (**A**) Photographs of mouse skin after 8 weeks of UV irradiation. Mice were treated with PBS, 40 μM G3CY-10, or 40 μM Vc in a microemulsion gel following UV exposure. The Sham group represents untreated mice without UV exposure. In the PBS group, mice received PBS in microemulsion gel post-UV exposure. (**B**,**C**), HE staining results of skin tissue, with the black arrow indicating the epidermal layer. The scale bar is 50 μm. Panel (**C**) quantifies the epidermal thicknesses for each group in panel (**B**), with values expressed as mean ± SEM from three independent experiments (n = 5). (**D**,**E**) Masson staining results of skin tissue, where the red area denotes muscle fibers and the blue area indicates collagen fibers. The scale bar is 50 μm. Panel (**E**) quantifies the positive area of collagen fibers, with values expressed as mean ± SEM from three independent experiments (n = 5). *, *p* < 0.05; **, *p* < 0.01; by unpaired *t* test.

As illustrated in Figure 6A, the skin of mice in the control group treated with a blank PBS microemulsion gel exhibited a dull appearance, increased thickness, rough texture with numerous wrinkles, and pronounced erythema. The administration of the G3CY-10 microemulsion gel significantly ameliorated UV-induced photoaging damage. Mice treated with G3CY-10 exhibited improved epidermal adhesion and a reduction in erythema, with effects comparable to those observed with an equivalent dose of Vc microemulsion gel (Figure 6A). Subsequent histopathological analysis indicated a significant reduction in epidermal thickness and sebaceous gland hyperplasia in the G3CY-10 group, with collagen fibers arranged in an orderly and moderately dense manner (Figure 6B,C), suggesting that G3CY-10 contributes to the maintenance of skin elasticity and firmness. Masson staining supported these findings, showing that collagen fibers in the G3CY-10 treatment group were more regularly arranged, more abundant, and exhibited more intense staining (25.60 ± 3.61%) compared to the PBS group (7.91 ± 0.73%) (Figure 6D,E), thereby indicating that G3CY-10 effectively inhibits the degradation of collagen fibers associated with photoaging. Interestingly, the G3CY-10 group and Vc exhibited comparable efficacy in mitigating photoaging-induced skin thickening and collagen fiber degradation, as illustrated in Figure 6C,E. The role of Vc in promoting collagen synthesis and preventing collagen degradation is well-documented in the literature. Numerous studies have highlighted Vc’s ability to enhance skin health by upregulating collagen production and reducing oxidative stress, both of which are critical in counteracting photoaging [20]. The potential of G3CY-10 to provide similar protective effects merits further investigation to establish its efficacy relative to vitamin C.

### 3.4. The Mechanism of G3CY-10 in Anti-Skin Photoaging Effects

**Figure 7:**
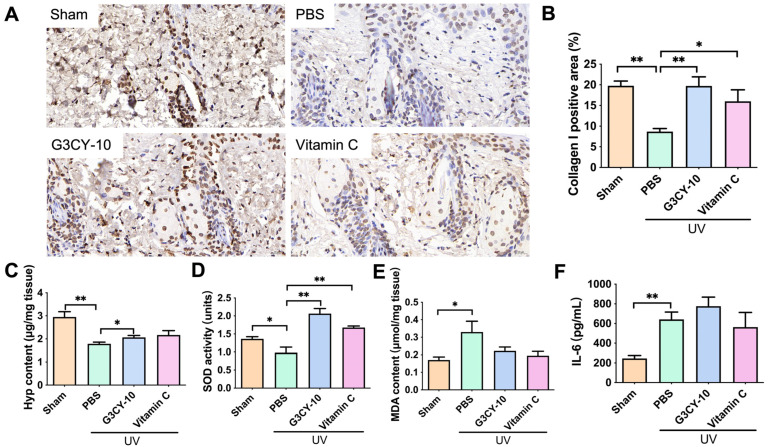
Effects of G3CY-10 on collagen metabolism, oxidative stress, and skin inflammation within a photoaging skin model. (**A**,**B**) Immunohistochemical staining was employed to assess the expression of Collagen I in skin tissue, with Collagen I appearing as a brown stain. The scale bar represents 20 μm. (**B**) The positive staining area for Collagen I in the skin tissue was quantified. The Sham group denotes the untreated mice without UV exposure, while the PBS group consists of mice treated with PBS in a microemulsion gel following UV exposure. Data are presented as mean ± SEM from three independent experiments (n = 7). (**C**) The expression levels of Hyp in skin tissue. Values represent mean ± SEM from three independent experiments (n = 6). (**D**,**E**) The levels of SOD (**D**) and MDA (**E**) in skin tissue. Skin specimens were precisely weighed and homogenized to produce tissue homogenates, from which supernatants were collected to determine SOD activity and MDA content. Data are presented as mean ± SEM from three independent experiments (n = 5). (**F**) The expression levels of the inflammatory factor IL-6 in skin tissue, with data shown as mean ± SEM from three independent experiments (n = 3). Statistical significance is indicated by *, *p* < 0.05; **, *p* < 0.01, as determined by an unpaired *t* test.

Skin photoaging is a complex pathological process initiated by UV radiation, encompassing a range of biological events such as inflammatory responses, oxidative stress, DNA damage, mitochondrial dysfunction, decreased collagen synthesis, extracellular matrix degradation, and autophagy dysregulation. In this study, we explored the anti-photoaging mechanism of G3CY-10 by concentrating on three critical aspects: collagen metabolism, oxidative stress, and skin inflammation. Initially, immunohistochemistry was utilized to evaluate the expression of type I collagen in skin tissues exposed to UV radiation. As depicted in Figure 7A,B, UV irradiation significantly diminished the expression of type I collagen, whereas treatment with G3CY-10 notably mitigated this reduction (19.71 ± 2.21% collagen I positive area in G3CY-10 group vs. 8.67± 0.75% in PBS group). Given that Hyp is a distinctive amino acid found in collagen, its concentration was quantified and multiplied by 7.46 to estimate total collagen levels. As demonstrated in Figure 7C, G3CY-10 treatment significantly elevated Hyp content (2.01 ± 0.08 μg/mg tissue) in comparison to the PBS group (1.79 ± 0.07 μg/mg tissue), corroborating the notion that G3CY-10 exerts anti-photoaging effects by inhibiting UV-induced collagen degradation.

Ultraviolet radiation can indirectly inflict damage on skin cell DNA by inducing oxidative stress, which may ultimately result in cellular necrosis. Consequently, mitigating oxidative stress emerges as a promising strategy for the prevention of skin photoaging. Our findings indicate that G3CY-10 significantly counteracted the UV-induced reduction in the activity of the antioxidant enzyme SOD, as illustrated in Figure 7D (2.06 ± 0.14 units vs. 0.98 ± 0.16 units in PBS group, *p* < 0.05). However, no significant effect was observed on the UV-induced elevation of MDA levels, a marker of lipid peroxidation (Figure 7E, *p* > 0.05). Additionally, skin photoaging is associated with the upregulation of pro-inflammatory cytokines. Following an 8-week treatment period, we evaluated the expression levels of inflammatory factors in murine skin. As depicted in Figure 7F, G3CY-10 did not significantly modify the inflammatory response elicited by UV exposure (*p* > 0.05). Collectively, these results suggest that G3CY-10 primarily exerts its anti-photoaging effects through the modulation of collagen metabolism and oxidative stress pathways, while exerting minimal influence on inflammatory responses.

## 4. Discussion

The efficacy of a microemulsion formulation is intrinsically dependent on the compatibility among its oil, surfactant, and cosurfactant components. To optimize this ternary system, we utilized the “maximization of pseudo-ternary phase diagram area” as the principal evaluation criterion. Anhydrous ethanol was chosen as the cosurfactant, and a “combination-elimination” screening strategy was employed to identify suitable oil and surfactant components. Ultimately, the lecithin/ethyl butyrate system was determined to be optimal. This selection was informed by both polarity matching (HLB = 10–12) and safety considerations: lecithin, an endogenous phospholipid, provides excellent biocompatibility, while ethyl butyrate offers low irritation and good solubility, facilitating the effective solubilization of hydrophobic peptides. Further optimization indicated that a Km value of 1:1 and a surfactant-to-oil ratio of 4:1 achieved the maximum water-loading capacity, closely aligning with the reported optimal Km range for lecithin-based microemulsions (0.8–1.2) [21]. The resulting formulation exhibited a particle size of 34.6 nm and demonstrated the Tyndall effect, signifying a monodisperse and thermodynamically stable O/W nanoemulsion [22].

Transdermal penetration studies have demonstrated a progressive, three-tiered enhancement in permeability. After 8 h, the aqueous solution remained confined to the epidermis, whereas the microemulsion gel penetrated the dermis within 0.5 h and achieved complete subcutaneous diffusion within 8 h. This enhanced permeability is attributable to three synergistic mechanisms: the size effect, where ~30 nm microemulsion particles rapidly traverse keratinocyte gaps (≤100 nm); the hydration effect, wherein the oil-in-water microemulsion, containing over 60% water, increases stratum corneum hydration by 30–40%, thereby reducing barrier resistance; and structural mimicry, as lecithin-derived phosphatidylcholine shares structural similarities with stratum corneum ceramides, facilitating its integration into intercellular lipid layers and inducing a “fluidization” effect [23]. Importantly, the microemulsion gel was formulated with P407, which enhances skin adhesion and minimizes formulation loss. P407, a widely used thickener and emulsifier, significantly improves the adhesion and stability of microemulsion gels, as corroborated by multiple studies [24]. Nanoemulsions and nanogels have been demonstrated to substantially enhance the skin permeability and stability of peptides, thereby augmenting their therapeutic efficacy [25,26]. Recent advancements in the integration of nanocarriers with microneedle technology have unveiled novel opportunities for the transdermal delivery of peptides, particularly showing considerable promise in the treatment of skin photoaging. Empirical evidence indicates that microneedle patches, as an innovative transdermal delivery system, are capable of effectively administering bioactive substances to alleviate the effects of photoaging [27]. The incorporation of microneedle technology can significantly enhance the efficacy of nanocarriers. Microneedle technology facilitates peptide transdermal penetration by creating microchannels in the skin [28]. Research has shown that the combination of nanocarriers with microneedle technology can markedly increase the transdermal delivery efficiency of insulin, achieving permeability rates hundreds of times greater than those achieved through passive diffusion [29]. Microneedle technology has exhibited distinct advantages in addressing skin photoaging. As an emerging transdermal delivery system, functional hyaluronic acid microneedles significantly improve photoaged skin conditions by promoting collagen regeneration and regulating oxidative stress [30]. These studies indicate that the integration of nanocarriers and microneedle technology not only opens new possibilities for transdermal delivery of peptides but also demonstrates significant potential in treating skin photoaging [31,32].

A UV-induced chronic photoaging mouse model was developed to replicate key histopathological characteristics of human skin aging, such as epidermal thickening, collagen degradation, erythema, and sebaceous gland hyperplasia. Administration of the G3CY-10 microemulsion gel at a concentration of 40 μM effectively ameliorated these pathological alterations, exhibiting efficacy comparable to that of the positive control, Vc. Notably, Vc is associated with potential irritation due to its acidic properties, whereas the G3CY-10 peptide system demonstrates greater physiological compatibility with the skin. Masson’s trichrome staining and IHC analysis indicated that G3CY-10 significantly enhanced the expression area of type I collagen and increased Hyp content relative to the PBS-treated model group, suggesting a regulatory effect on collagen degradation. This mechanism is consistent with existing literature on YKL-40, which is known to inhibit matrix metalloproteinase-1 activity and promote the formation of type I collagen fibers, thereby mitigating collagen breakdown [33]. These findings support the hypothesis that G3CY-10 primarily elevates collagen content through the inhibition of degradation rather than the promotion of synthesis.

In light of the complex mechanisms involved in photoaging, this study focused on two principal phenotypes: collagen depletion and oxidative stress. The compound G3CY-10 was found to be effective in reversing UV-induced depletion of superoxide dismutase (SOD), although it did not significantly influence malondialdehyde (MDA) levels or inflammatory cytokines such as interleukin-6 (IL-6). This observation suggests that the antioxidant properties of G3CY-10 may be due to the preservation of enzyme activity rather than the inhibition of lipid peroxidation. Silkworm pupal peptides (SPPs) have shown considerable efficacy in ameliorating photoaging. Evidence indicates that SPPs effectively slow down skin photoaging by markedly reducing excessive reactive oxygen species (ROS) and MDA, while concurrently restoring antioxidant enzyme activity [34]. This mechanism is consistent with the direct antioxidant action of the G3CY-10 peptide in delaying photoaging. Furthermore, SPPs inhibit collagen degradation, which corresponds to another critical mechanism of the G3CY-10 peptide. Additionally, the combination of collagen and epigallocatechin gallate (EGCG) demonstrates a synergistic effect in preventing UVB-induced photoaging of the skin [35]. Research indicates that this combination can restore the depletion of UVB-induced antioxidant enzymes, such as superoxide dismutase (SOD) and glutathione peroxidase (GSH-Px), while significantly mitigating collagen degradation. This mechanism is consistent with the action of the G3CY-10 peptide, which prevents SOD inactivation and collagen degradation. The critical role of antioxidant activity in protecting against skin photodamage has been extensively documented. Apigenin, a flavonoid compound, exhibits notable antioxidant and anti-inflammatory properties, restoring UV-induced skin cell vitality and reducing collagenase MMP-1 expression, thereby protecting the skin’s collagen matrix [36]. Similarly, studies on the tripeptide ACQ reveal its exceptional antioxidant activity, which protects skin cells from oxidative stress damage and increases epidermal stem cell quantity by regulating integrin α6 [37]. This antioxidant mechanism parallels the action mechanism of G3CY-10 peptide, further endorsing its application in photoprotection against skin photoaging. Regarding the inhibition of collagen degradation, collagen peptides promote procollagen synthesis by activating the TGF-β/Smad pathway and inhibit the expression of MMP-1 and MMP-3, thereby preventing collagen breakdown [38]. This mechanism aligns with the action of the G3CY-10 peptide, indicating its potential in maintaining skin structural integrity. Research on SOD enzyme activity maintenance indicates that antioxidant peptides can protect skin from photoaging by restoring SOD and glutathione peroxidase (GSH-Px) levels, thereby reducing UV-induced oxidative stress [39]. Hydrogen bonds play a crucial role in maintaining protein structural integrity and functional activity. By donating hydrogen bonds, peptides can effectively interact with SOD, thereby stabilizing its conformation and enhancing its enzymatic activity [40]. Those mechanisms align with the inhibitory effect of G3CY-10 peptide on SOD enzyme inactivation. Additionally, the observed suppression of inflammatory signaling by G3CY-10 should be viewed as an advantage rather than a limitation, as it mitigates the immunosuppressive risks associated with prolonged use of topical corticosteroids or NSAIDs, underscoring the mild and low-side-effect profile of peptide-based therapies. Future research may employ transcriptomic analyses to investigate whether G3CY-10 influences collagen metabolism and antioxidant gene expression through the TGF-β/Smad or Nrf2/ARE pathways.

G3CY-10 micelles exhibit significant anti-photodermaging properties, thereby establishing a critical theoretical basis for potential therapeutic applications in humans. Nevertheless, the safety and efficacy of this formulation must be prioritized during its clinical translation. The use of nanoemulsion technology in peptide drug delivery has been extensively studied. As an emerging drug delivery system, nanoemulsions have attracted considerable attention due to their ability to enhance drug solubility and bioavailability. Research indicates that nanoemulsions can effectively deliver peptide drugs to target sites, thereby improving therapeutic efficacy. For example, DALDA peptide analogs administered via oil-in-water nanoemulsions to the central nervous system have demonstrated excellent analgesic effects and safety profiles [41]. Moreover, innovative applications of nanoemulsions in oral administration, injections, and ophthalmic treatments have expanded the possibilities for peptide drug delivery [42]. In the context of treating skin photoaging, addressing the formulation challenges and safety considerations of G3CY-10 nanoemulsions remains a key research priority. The distinctive characteristics of nanoemulsions—such as their high surface area, small droplet size, and exceptional physical stability—render them ideal candidates for drug delivery systems [43,44]. Nonetheless, a comprehensive understanding of the foundational principles underlying nanoemulsion preparation and functionalization remains essential for their successful clinical application [44]. In the context of transdermal delivery, nanoemulsions have the potential to enhance therapeutic efficacy by increasing drug bioavailability through improved skin permeability [45,46]. Concerning the safety profile of nanomicelles, existing studies suggest that oral administration is generally safe, whereas high-dose injections may induce toxic reactions [47]. Therefore, thorough safety assessments are imperative during clinical development to ensure their suitability for human use. Furthermore, environmental toxicity presents a significant issue. Research indicates that certain nanomicelles may exert toxic effects on environmental organisms, highlighting the necessity for formulation strategies aimed at minimizing ecological impact [48]. In the subsequent phase of clinical development, it is advisable to undertake preclinical and clinical trials of G3CY-10 nanoemulsions to substantiate their efficacy and safety in addressing skin photoaging. By optimizing the formulation and delivery system of the nanoemulsion, its penetration into the skin and drug release efficiency can be enhanced [49,50]. Moreover, incorporating recent advancements in nanoemulsion technology, such as nanoemulsion gels and microneedle delivery systems, could further expand its potential for topical applications [51,52].

## 5. Conclusions

In conclusion, the G3CY-10 peptide was successfully delivered transcutaneously via a lecithin/ethyl butyrate oil-in-water microemulsion gel, exhibiting significant collagen-protective and antioxidant effects in a UVB-induced photoaging mouse model, while maintaining a favorable safety profile. This study not only validates the potential of microemulsion gels as effective transdermal delivery systems but also provides a replicable technical framework and theoretical foundation for the development of peptide-based topical formulations aimed at combating photoaging.

## Figures and Tables

**Table 1:** Preliminary screening of formulations involving different oil phases and mixed surfactants.

	Surfactant/Co-Surfactant	Lecithin/Anhydrous Ethanol	Span80/Anhydrous Ethanol	Tween80/Anhydrous Ethanol
Oil Phase				
castor oil		−	+	+
ethyl butyrate		+	−	+

“−” signifies that the mixture of mixed surfactants and the oil phase fails to form a microemulsion, whereas a “+” indicates successful microemulsion formation upon mixing.

## Data Availability

The original contributions presented in this study are included in the article. Further inquiries can be directed to the corresponding authors.

## Abbreviations

The following abbreviations are used in this manuscript:

UV	Ultraviolet
Km	The mass ratio of surfactant to cosurfactant
TEM	Transmission electron microscopy
FITC	Fluorescein isothiocyanate
MED	Minimal erythema dose
HE	Hematoxylin and eosin
IHC	Immunohistochemistry
PBS	Phosphate-buffered saline
BSA	bovine serum albumin
DAB	Diaminobenzidine
ELISA	Enzyme-linked immunosorbent assay
SOD	Superoxide dismutase
MDA	Malondialdehyde
Hyp	Hydroxyproline
Vc	Vitamin C
